# Breaking the Barrier: Strategies for Mitigating Shuttle Effect in Lithium–Sulfur Batteries Using Advanced Separators

**DOI:** 10.3390/polym15193955

**Published:** 2023-09-30

**Authors:** Yingbao Zhu, Zhou Chen, Hui Chen, Xuguang Fu, Desire Emefa Awuye, Xichen Yin, Yixuan Zhao

**Affiliations:** 1 School of Mechanical and Power Engineering, Nanjing Tech University, Nanjing 211800, China; 202161207212@njtech.edu.cn (Y.Z.); 202261107032@njtech.edu.cn (X.Y.); 202021106006@njtech.edu.cn (Y.Z.); 2 Jiangsu Zhongneng Polysilicon Technology Development Co., Ltd., Xuzhou 221000, China; liuhui@gcl-power.com (H.C.); fuxuguang@gcl-power.com (X.F.); 3 Department of Minerals and Materials Engineering, University of Mines and Technology, Tarkwa 03123, Ghana; deawuye@umat.edu.gh

**Keywords:** lithium–sulfur batteries, shuttle effect, separator, separator modification

## Abstract

Lithium–sulfur (Li-S) batteries are considered one of the most promising energy storage systems due to their high theoretical capacity, high theoretical capacity density, and low cost. However, challenges such as poor conductivity of sulfur (S) elements in active materials, the “shuttle effect” caused by lithium polysulfide, and the growth of lithium dendrites impede the commercial development of Li-S batteries. As a crucial component of the battery, the separator plays a vital role in mitigating the shuttle effect caused by polysulfide. Traditional polypropylene, polyethylene, and polyimide separators are constrained by their inherent limitations, rendering them unsuitable for direct application in lithium–sulfur batteries. Therefore, there is an urgent need for the development of novel separators. This review summarizes the applications of different separator preparation methods and separator modification methods in lithium–sulfur batteries and analyzes their electrochemical performance.

## 1. Introduction

With the rising demand for electric vehicles and portable electronic devices, it is important to research and develop energy storage systems with high energy density, low cost, and extended service life [1]. Traditional lithium batteries are widely used due to their high working voltage, long cycle life, and good stability [2]. However, the high production cost, theoretical specific capacity, and low energy density of the electrode material make it difficult for traditional lithium batteries to meet the increasing market scale [3]. Lithium-sulfur (Li-S) batteries are regarded as one of the energy storage systems with great potential due to its low production cost, high theoretical specific capacity and energy density, abundant S elements in nature, and low toxicity [4]. They are also facing great challenges, such as the poor conductivity of S elements, the volume expansion of the cathode material, and the shuttle effect caused by lithium polysulfide in the commercial development of Li-S [5,6]. Solving the above problems is crucial for the commercialization of lithium-sulfur batteries.

**Figure 1:**
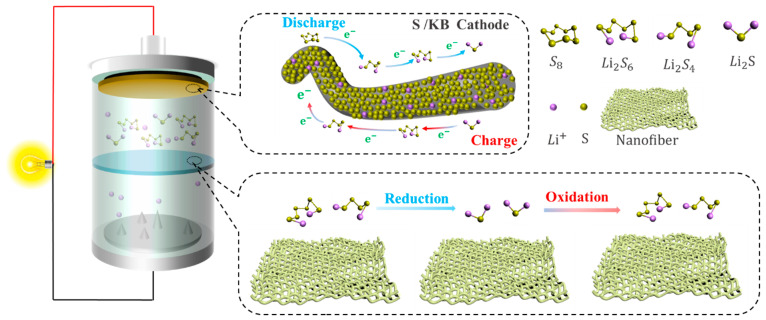
Lithium–sulfur battery model separator.

Li-S batteries are composed of an anode, a cathode, an electrolyte, and a separator, as shown in Figure 1, which represents a schematic diagram of a typical Li-S battery, in which the separator plays a key role in solving the shuttle effect of lithium polysulfide [7,8]. The shuttle effect occurs because lithium polysulfide is easily soluble in the electrolyte and moves between the cathode and the anode, resulting in irreversible volume loss [9]. Therefore, studying a separator with excellent performance is very important in order to solve the shuttle effect.

**Figure 2:**
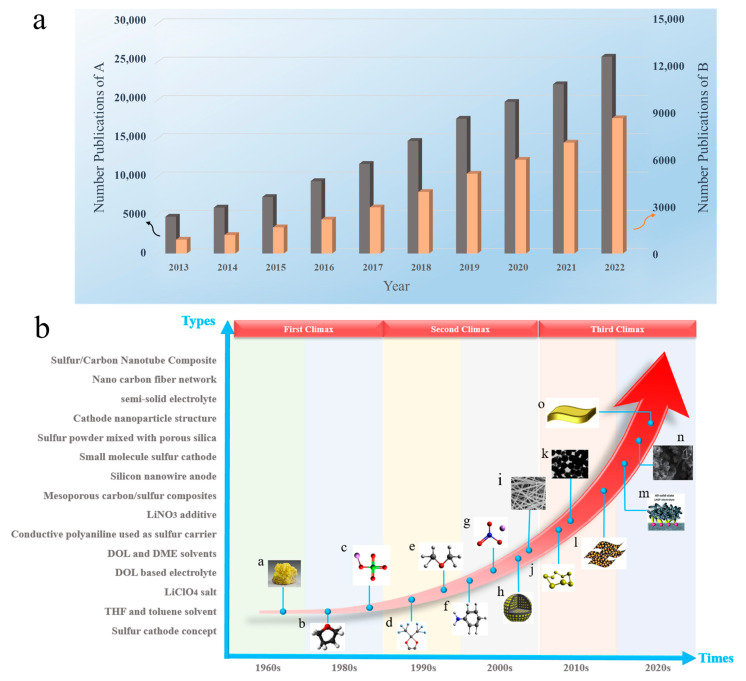
Lithium–sulfur battery development history: (**a**) Publications A and B in the past decade by searching for “lithium–sulfur battery separator” and “lithium–sulfur battery” as “keywords” on the Google Scholar website. (**b**) The development history of lithium–sulfur batteries: (a) sulfur cathode concept; (b) THF and toluene solvent; (c) LiClO_4_ salt; (d) DOL-based electrolyte; (e) DOL and DME solvents; (f) conductive polyaniline used as sulfur carrier; (g) LiNO_3_ additive; (h) mesoporous carbon/sulfur composites; (i) silicon nanowire anode; (j) small molecule sulfur cathode; (k) sulfur powder mixed with porous silica; (l) cathode nanoparticle structure; (m) semi-solid electrolyte; (n) nano carbon fiber network; (o) sulfur/carbon nanotube composite [12,13,14,15,16]. Reproduced with permission from the Particle & Particle Systems Characterization and Journal of Materials Chemistry A and Nano Research and Energy and Royal Society Of Chemistry.

As shown in Figure 2a, the gray color in the histogram represents the number of publications (A), corresponding to the left axis. The orange histogram represents Publication B, and the quantity corresponds to the right axis. The number of publications on Li-S separators has increased year by year, and this shows that Li-S separators have been a research hotspot in this field.

At present, commercial Li-S separators mainly consist of PP and PE separators [10], which are widely used in Li-S due to their good chemical stability and good mechanical strength. However, this type of separator has a weak ability to inhibit the shuttle effect of polysulfides, poor affinity with electrolytes, and low porosity, resulting in poor battery performance, so this type of olefin separator is not well used in Li-S [11].

To suppress the shuttle effect in Li-S, it is necessary to study a new type of separator with good performance or to modify the original commercial separator. Figure 2b shows the main development history of lithium–sulfur batteries. In recent years, there has been a proliferation of comprehensive reviews on lithium–sulfur battery separators. However, these reviews tend to be confined to specific fabrication techniques such as electrospinning, deposition methods, or filtration processes. In contrast, this study not only encompasses an overview of conventional methods for fabricating lithium–sulfur battery separators and their applications in lithium–sulfur batteries, but also extends its scope to include composite fabrication processes and their potential applications in lithium–sulfur batteries. This paper reviews the lithium–sulfur battery separators prepared by different preparation or modification methods in recent years, which inhibit the shuttle effect of polysulfides and improve the electrochemical performance. Finally, future research directions involving lithium–sulfur batteries are analyzed, and the prospect of building high-performance lithium–sulfur batteries is introduced.

## 2. The Working Principle and Problems of Li-S

### 2.1. Working Mechanism of Li-S

The electrochemical reactions that occur in Li-S are different from traditional lithium batteries [17], and the redox reaction during the charging and discharging of Li-S involves the conversion between different valence ions of the S atom, making it more complex than traditional lithium-ion batteries [18,19]. Lithium–sulfur batteries generally consist of a lithium metal negative electrode, an organic liquid electrolyte, and a sulfur–carbon composite positive electrode [20]. Taking a positive electrode material such as sulfur elemental as an example, during discharge, the lithium metal of the anode is oxidized to generate electrons and lithium ions, and the lithium ions diffuse to the cathode through the electrolyte. At the same time, the electrons move to the sulfur element of the cathode through the external circuit, the sulfur element is reduced to S^2−^, and S^2−^ and Li^+^ generate Li_2_S at the cathode [21]. During the discharge process, when the sulfur element in the cathode reacts completely with the lithium ion, the total equation of the electrochemical reaction is as follows:

(1) $$S_{8}+16{Li}^{+}+16e^{-}↔8{Li}_{2}S$$

**Figure 3:**
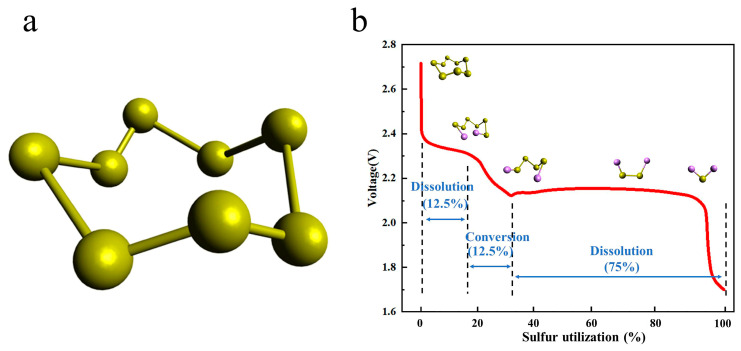
(**a**) Schematic diagram of the spatial structure of elemental sulfur. (**b**) Red is the discharge curve of lithium-sulfur battery, showing the sulfur electrode utilization.

As shown in Figure 3a, the spatial structure of elemental sulfur is formed by eight sulfur atoms connected by covalent bonding, so there are multiple cleavage and bonding sites of S-S bonds in sulfur elements, resulting in more complex redox reactions in the charging and discharging process of Li-S [20,22]. At present, there are many studies on the S conversion of lithium–sulfur battery charging and discharging process [22,23]. According to the measurement of the electrolyte mass spectrometry (LC/MS) of different charges and discharges, the reduction reaction of elemental sulfur is carried out in multiple steps, and there are a variety of different intermediate products, such as Li_2_S_8_, Li_2_S_6_, Li_2_S_4_, Li_2_S_2_, Li_2_S_2_, and Li_2_S [24].

When discharging, the electrochemical reaction is as formulated (2)–(6) [17]:

(2) $$S_{8}+2e^{-}+2{Li}^{+}\to {Li}_{2}S_{8}$$

(3) $$3{Li}_{2}S_{8}+{2e}^{-}+{2Li}^{+}\to {4Li}_{2}S_{6}$$

(4) $${2Li}_{2}S_{6}+{2e}^{-}+{2Li}^{+}\to {3Li}_{2}S_{4}$$

(5) $${Li}_{2}S_{4}+{2e}^{-}+{2Li}^{+}\to {2Li}_{2}S_{2}$$

(6) $${Li}_{2}S_{2}+{2e}^{-}+{2Li}^{+}\to {2Li}_{2}S$$

As can be seen in Figure 3b, it can be seen from the discharge curve of the Li-S that there are two discharge platforms in the discharge process [25]. The high discharge platform is between 2.3~2.4 V; at this time, the S8 product is a variety of soluble long-chain lithium polysulfide Li_2_S_x_ (4 ≤ x ≤ 8) [26]. The low discharge platform is between 1.8~2.1 V; at this time, the corresponding long-chain lithium polysulfide Li_2_S_x_ (4 ≤ x ≤ 8) is further reduced to short-chain lithium polysulfide Li_2_S_x_ (1 ≤ x ≤ 4) [27] (Wu et al. [28]). According to experimental calculations, the utilization rate of the active material of the high discharge platform is only 25%, and the theoretical capacity of the process is 418 mAh g^−1^. The active material utilization rate of the low discharge platform is 75%, and the theoretical capacity of the process is 1254 mAh g^−1^. At the low discharge platform, due to the growth of lithium dendrites and phase transition processes, the reaction kinetics of this process slow down, and the actual discharge-specific capacity of the battery is lower than the theoretical capacity, so the actual specific capacity of the Li-S is lower than the theoretical specific capacity throughout the entire discharge process [29,30,31,32].

When charging, there is only one noticeable platform of about 2.4 V [33]. Electrochemical reactions can be described by Formulas (7) and (8):

(7) $${2S}_{4}^{2-}-{2e}^{-}\to S_{8}^{2-}/S_{8}$$

(8) $$S_{8}^{2-}/S_{6}^{2-}-{2e}^{-}{=S}_{8}$$

### 2.2. The Existing Problems of Li-S

#### 2.2.1. Cathode

**Figure 4:**
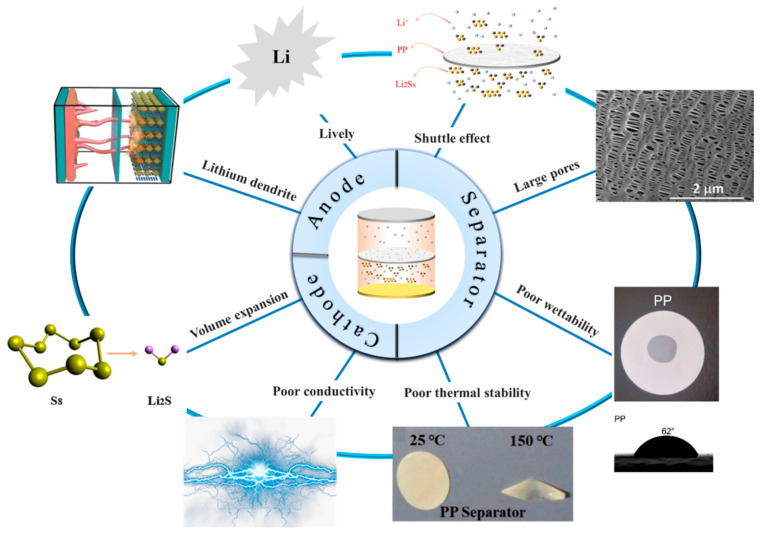
Existing problems with lithium–sulfur batteries [61]. Reproduced with permission from the Polymers.

Because sulfur resources are abundant, non-toxic, and non-polluting; the theoretical specific capacity is 1675 mAh g^−1^; and they have low prices and other advantages, sulfur was selected as the cathode material in Li-S batteries [34,35,36]. However, problems such as low conductivity, volume expansion during electrochemical reactions, and shuttle effect lead to low coulombic efficiency, and the actual specific capacity is much lower than the theoretical specific capacity, in addition to poor cycle stability [37], as shown in Figure 4. Among these, the shuttle effect [38,39] is the most important factor for the short cycle life and low specific capacity of Li-S. The solution to these problems is crucial for the commercialization of Li-S. Wang et al. [40] synthesized sulfur particles coated with PEG surfactants and wrapped them in carbon-black-decorated graphene oxide flakes in a simple assembly process. The graphene–sulfur composite showed a relatively stable specific capacity of about 600 mAh g^−1^ and attenuation of less than 15% in 100 cycles. Yang et al. [36] used boron-doped porous carbon material as the host material of the S cathode. B-doped carbon material exhibits higher conductivity than pure porous carbon. At 0.25 C, the S/B doped carbon cathode can provide a higher initial capacity of 1300 mAh g^−1^ compared to the cathode based on pure porous carbon. The cycle stability and rate capability are also improved.

#### 2.2.2. Separator

As a vital part of Li-S, separators play a great role in the performance of Li-S [41]. During the charging and discharging process of lithium–sulfur batteries, the separator does not directly participate in the electrochemical reaction of mutual conversion between polysulfides [42]. However, the wettability, thermal stability, mechanical properties, porosity, liquid absorption rate, and other properties of the separator affect the specific capacity and cycle life of the Li-S, as shown in Figure 4. As a separator for Li-S, it is not only necessary to maintain the advantages of wettability, thermal stability, mechanical properties, porosity, and liquid absorption rate [43], but also to effectively suppress the shuttle effect [44].

With the development of clean energy, traditional separators such as polypropylene, polyethylene, and polyimide are widely employed in lithium-ion batteries. However, due to the distinct operational mechanisms of lithium-ion batteries and lithium–sulfur batteries, these traditional separators present certain challenges when applied in lithium–sulfur battery systems. Primarily, the major issue lies in the inability of traditional separators to impede the shuttle effect of polysulfides. Owing to the relatively large pores in conventional separators, polysulfides can readily permeate, leading to diminished battery capacity and low Coulombic efficiency. Secondarily, traditional polypropylene and polyethylene separators exhibit inadequate resistance to high temperatures, posing safety risks under elevated operating temperatures. Polyimide separators suffer from suboptimal mechanical properties, rendering them susceptible to lithium dendrite penetration and associated safety concerns.

#### 2.2.3. Anode

The weight density is low, and the theoretical specific capacity is 3860 mAh g^−1^ higher [45]. Due to its low reduction potential [46,47] and other advantages, lithium metal was chosen as the electrode material for Li-S. Metallic lithium is used as an anode in Li-S [48] due to the growth of lithium dendrites [40,49,50,51]. Lithium dendrites easily pierce the separator, resulting in potential safety hazards in the circuit. Lithium metal itself has strong activity and can easily have side reactions with electrolytes, resulting in low battery cycle life and other problems that limit the commercial development of Li-S, as shown in Figure 4. At present, in response to the existing problems of anode lithium metal, a large number of researchers focus on electrolyte additives and lithium anode surface to form a protective film (artificial SEI film) [52,53,54] and explore several aspects of other materials that can replace metal lithium anodes. To inhibit the growth of lithium dendrites on the anodes of Li-S, Guo et al. [55] used VC-LiNO as an electrolyte additive and an efficiency of lithium plating/stripping up to 100%, resulting in a uniform and stable SEI separator with high ionic conductivity (Li et al. [56]). The artificial Li_3_PO_4_ SEI layer was prepared, which effectively inhibited the growth of lithium dendrites and had good chemical stability (Jan et al. [57]). Si-C and hard carbon anodes were prepared, which improved the cycle stability of the battery. The Coulombic efficiency of more than one thousand cycles was higher than 99%, and the attenuation of each cycle capacity was only 0.08%.

#### 2.2.4. Electrolyte

Electrolytes, as a pivotal constituent bridging the positive and negative electrodes, wield substantial influence on the transport of lithium ions, thereby exerting a direct impact on the performance of lithium–sulfur batteries. Electrolytes can be classified into two categories: liquid electrolytes and solid electrolytes.

Presently, liquid electrolytes find extensive application in lithium–sulfur batteries, primarily owing to their facile synthesis, high ionic conductivity, and favorable chemical stability. Nevertheless, liquid electrolytes are not without their challenges. Firstly, at the positive electrode, liquid electrolytes tend to dissolve polysulfides, which subsequently diffuse through the electrolyte to the negative electrode, leading to diminished Coulombic efficiency and corrosion of the lithium negative electrode. Secondly, liquid electrolytes typically comprise flammable organic solvents, thus posing safety concerns when exposed to elevated temperatures. In response to these issues associated with liquid electrolytes, a substantial body of researchers is actively engaged in addressing these challenges. This includes the modification of electrolyte composition, the exploration of novel multi-component solvents, and the incorporation of functional additives [58].

The application of solid-state electrolytes effectively mitigates the “shuttle effect” caused by the dissolution of polysulfides while also eliminating flammable organic solvents, thereby significantly enhancing the safety of lithium–sulfur batteries. However, current lithium–sulfur batteries employing solid-state electrolytes still face challenges such as low ionic conductivity and high interfacial impedance, which impede the further advancement of solid-state electrolyte technology. To address these issues, ongoing research efforts are predominantly focused on the development of novel functional materials and the exploration of inorganic fillers as strategies to tackle these challenges.

#### 2.2.5. Binders

Binders, constituting an integral part of the sulfur cathode in lithium–sulfur batteries, serve the crucial function of ensuring effective electrochemical contact among the conductive agent, sulfur, and current collector. Moreover, they mitigate volumetric variations of active materials during cycling. Binders play a pivotal role in lithium–sulfur batteries, and the commonly utilized types encompass polymeric, bio-based, and inorganic binders. Challenges regarding binders encompass inadequate mechanical properties and the detachment of electrode materials during cycling, consequently leading to a diminished cycling lifespan and reduced stability of the battery. In the future, bio-based binders exhibiting natural, renewable, and superior adhesive properties hold substantial promise for advancement, thus augmenting lithium–sulfur batteries [59].

#### 2.2.6. Current Collector

Aluminum foil has found widespread application as a current collector for sulfur cathodes. However, at elevated temperatures, aluminum and sulfur can undergo reactions, which may pose safety concerns. Presently, the use of carbon-coated aluminum foil is employed to mitigate direct contact between sulfur and aluminum foil, thereby enhancing the safety of lithium–sulfur batteries. The presence of carbon coatings effectively improves the adhesion between the active materials and the current collector, while simultaneously enhancing electrical conductivity [60].

## 3. Methods

With the development of science and technology, separator technology has also been continuously developed and updated, and the methods of preparing polymer separators have also been diversified. Different preparation methods can be selected according to the material properties and separator application direction. The battery separator film is closely related to the energy density, stability, and cycle life of the battery [62]. A battery separator composed of nanofibers has the advantages of a large specific area and high porosity [63], which has attracted close attention. There are many methods of separators, but electrospinning [64], vacuum filtration [65], wet spinning [66], the coating method [67,68], the in situ growth method [69], and atomic layer deposition [70] are more commonly used to prepare separators.

### 3.1. Electrospinning

**Figure 5:**
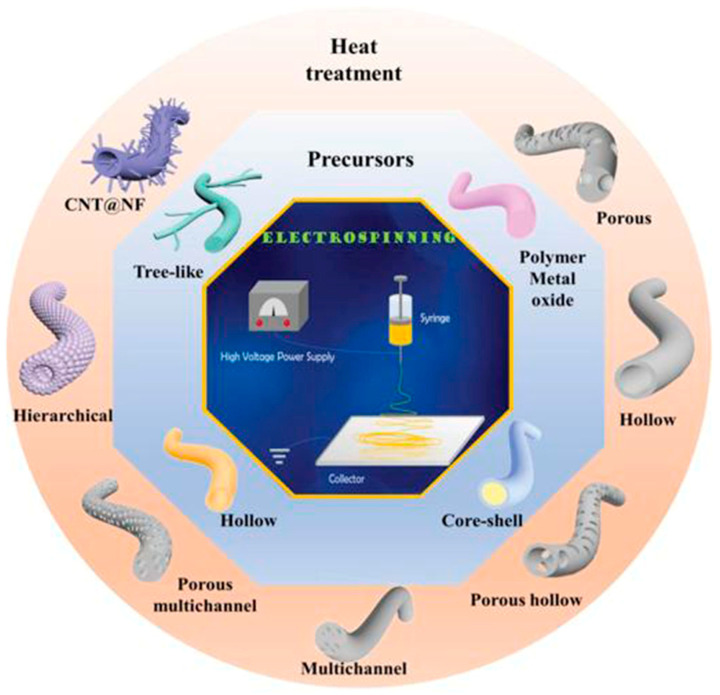
The electrospinning technology has prepared a variety of nanostructured fibers [2]. Reproduced with permission from the Advanced Functional Materials.

Electrospinning is a novel technique for preparing nanofiber separators, the principle of which is as follows:: under the action of a high-voltage electrostatic field, the polymer forms a Taylor cone when it flows out of the needle, and a continuously charged jet is ejected and deposited on the collector as nonwoven nanofibers [71]. The nanofibers prepared by electrospinning can reach the nanometer diameter [64,72], and the prepared nanofiber separator has the advantages of high porosity, large specific surface area, and small pore size [73]. As shown in Figure 5, electrospinning technology has prepared a variety of nanostructured fibers. The electrospinning method has received great attention in the field of battery separators in recent years.

### 3.2. Vacuum Filtration

The vacuum filtration method is a new method for preparing separators using the interfacial composite method, which has the advantages of convenient operation, a simple process, and no specific process equipment. Its working principle is that the film-forming material is uniformly dispersed in the solvent, and then under vacuum filtration conditions, the film-forming material is deposited on the substrate due to the flow function of solvent molecules. The disadvantage is that due to the limitations of process conditions, the structure of the produced nanofibers is uncontrollable and unsuitable for large-scale mass production [74].

### 3.3. Wet Spinning

The nanofiber non-woven separator prepared by the wet laying method has high porosity and good affinity with electrolytes [75]. The principle is that different polymer fibers are used to separate matrix fibers and adhesive fibers, which are continuously randomly laid on the screen belt after mixing in a water-soluble suspension, sent to a ventilation drying box to heat and soften the bonded fibers, and then calendered by a heating drum to obtain a non-woven film [62]. The disadvantages are that the structure and properties of the nanofibers prepared by this method are limited by the type and composition of the polymer in the suspension, and the operation is more complicated [76].

### 3.4. Coating Method

The coating method is one of the most commonly used modification methods. A layer of modified material is coated on one side of the separator by means of physical methods, and polysulfides are adsorbed by the modified material to improve the shortcomings of the separator. Because the coating method is simple to operate, relatively low in cost, can be used for large-area separator modification, and is suitable for industrial production, the coating method is widely used in the modification of lithium–sulfur battery separators. However, the coating method has problems such as difficulty in accurately controlling the thickness of the separator. The viscosity of the material has an impact on the coating effect, and some polymer materials are not suitable for the preparation of the coating method.

### 3.5. In Situ Growth Method

The in situ growth method is a relatively novel method of separator modification. Compared with other modification methods, this method can effectively generate a lightweight barrier on one side of the separator and avoid excessive thickness of the separator; in addition, the in situ growth method can achieve efficient use of materials, thereby reducing costs. However, the current in situ growth method is still in the experimental research stage. In addition, the modification process is relatively complicated, and it is difficult to achieve mass production.

### 3.6. Atomic Layer Deposition (ALD)

Atomic layer deposition is a method in which substances can be plated on the surface of the substrate, layer by layer, in the form of a single-atom film. It has similarities to chemical vapor deposition [77]. Its working principle is a method in which a gas precursor pulse alternately enters the reactor and chemically adsorbs and reacts on the deposition matrix to form a deposition film. A typical ALD cycle consists of two self-limiting semi-reactions [78], and it is this reaction property that allows the separator’s thickness to be precisely controlled. However, the atomic layer deposition method has disadvantages such as high requirements for reaction conditions, a complicated production process, expensive equipment, and a slow deposition rate.

## 4. Application of Separators in Lithium–Sulfur Batteries

As is evident from the preceding discussion, the separator plays a crucial role in influencing the electrochemical performance of lithium–sulfur batteries. In the subsequent section, we will delve into the utilization of separators fabricated through various methods within the context of lithium–sulfur batteries. The interlayer, being an integral component of the separator, typically resides between the positive electrode and the separator. Its exceptional attributes, such as the heightened surface area and excellent electronic conductivity, facilitate the diffusion of lithium ions and the conduction of electrons. Moreover, we will expound upon the application of interlayers prepared via different techniques in lithium–sulfur batteries.

### 4.1. Electrospinning

#### 4.1.1. Separator

**Figure 6:**
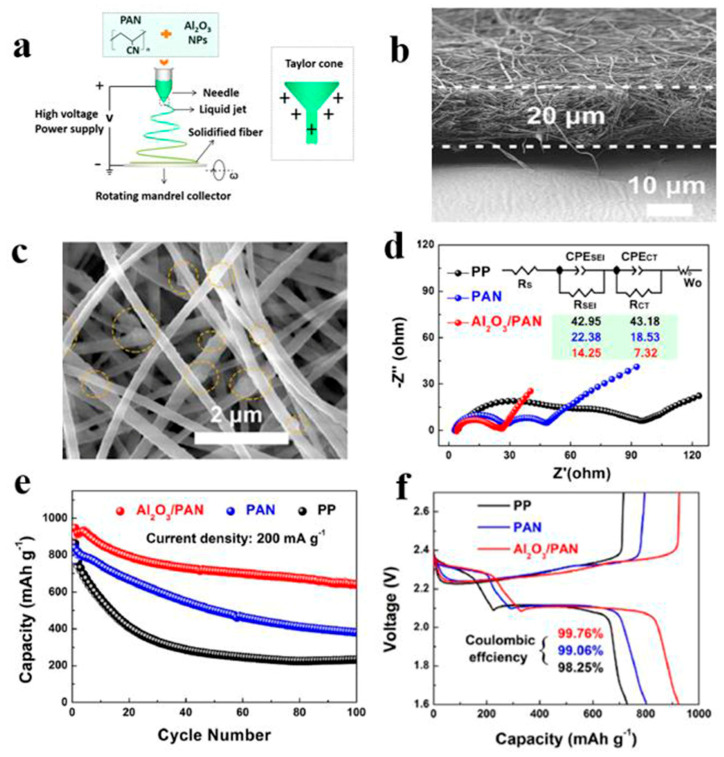
Application of an electrospinning separator in lithium−sulfur batteries: (**a**) Preparation process. (**b**) Cross−sectional morphologies of Al_2_O_3_/PAN separator prepared by electrospinning. (**c**) The SEM image shows Al2O3 distributed on the fiber. (**d**) EIS curve. (**e**) Cycle curve. (**f**) Charge−discharge curve [80]. Reproduced with permission from the Electrochimica Acta.

In Li-S, the separator is required to suppress the shuttle effect, good electrolyte wettability, and good ionic conductivity [79]. Nanofiber separators prepared using the electrospinning technique exhibit extremely fine fibers, with exceptionally high specific surface areas. Additionally, these separators possess a notably high level of porosity, a feature inherent to their unique fabrication process. Owing to their elevated specific surface areas and porosity, such separators can provide a greater quantity of electrolytes during the charge and discharge processes of lithium–sulfur batteries. This augmentation of reaction sites enhances electrochemical reactions. In recent years, numerous researchers have achieved significant advancements by incorporating modified materials onto the fiber surface, aiming to adsorb polysulfides and mitigate the shuttle effect, thereby improving the cyclic lifespan and practical specific capacity of the batteries (Guo et al. [80]). A PAN film doped with Al_2_O_3_ particles was prepared using the electrospinning method and studied as a separator for Li-S. Figure 6 shows the preparation process of the separator and the excellent electrochemical performance of the lithium–sulfur battery using the separator. It can be seen from the figure that Al_2_O_3_ particles attached to the PAN nanofibers, and the separator exhibited strong chemical interaction, which blocked polysulfides from passing through the separator and inhibited the shuttle effect. The PAN/Al_2_O_3_ separator was thermally stable at 200 °C. The lithium–sulfur battery with this separator exhibited low resistivity (RSEI = 14.25 Ω, RCT = 7.32 Ω). Under a 200 mAg constant current charge and discharge, the remaining capacity after 100 cycles was 639 mAh/g (compared with pure PAN and PP separator batteries, which achieved only 380 mAh/g and 233 mAh/g), and the Coulombic efficiency was 99.76%.

In comparison to traditional PP separator separators, electrospun nanofiber separators have garnered considerable attention due to their exceptional specific surface areas and extensive porosity. They exhibit unparalleled advantages in terms of electrolyte absorption and ion transport, positioning them as a focal point in research for potential battery separators.

#### 4.1.2. Interlayer

**Figure 7:**
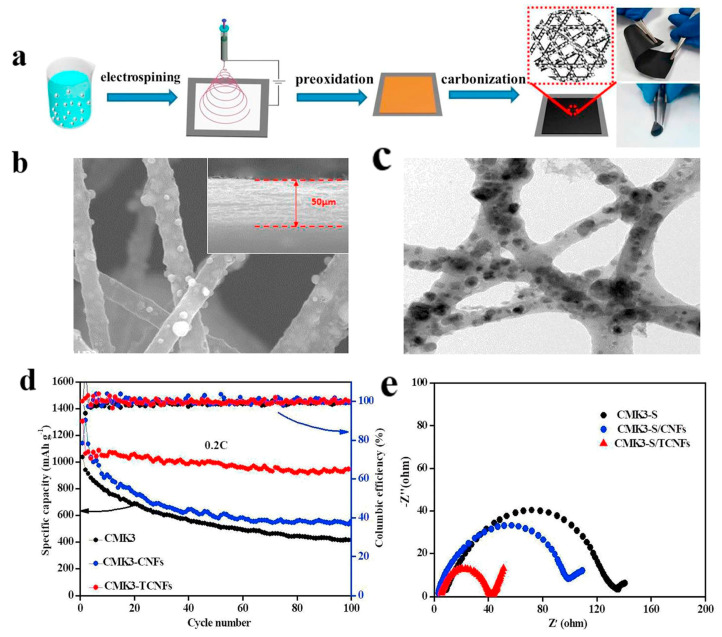
Application of electrospinning interlayer in lithium−sulfur batteries: (**a**) interlayer preparation process; (**b**) SEM images of TCNFs; (**c**) TEM images of TCNFs; (**d**) cycling stability of CMK3−S, CMK3-S/CNFs, and CMK3-S/TCNFs interlayers at 0.2 C for 100 cycles; (**e**) EIS curve [81]. Reproduced with permission from the Chemical Engineering Journal.

A highly conductive electrospun nanofiber interlayer is introduced between the positive electrode and the separator to effectively improve the performance of lithium–sulfur batteries. Electrospun carbon nanofibers, owing to their excellent electrical conductivity, serve to reduce internal resistance within batteries, thus enhancing the rates of electron and ion transport. Additionally, carbon nanofibers exhibit Van Der Waals interactions with polysulfides, facilitating their adsorption. Guo et al. [81]. utilized electrospinning technology to prepare a Ti_4_O_7_/C nanofiber (TCNF) interlayer. The incorporation of carbon nanofibers in this interlayer offered several advantages, including a large specific surface area and high electrical conductivity, which significantly enhanced the conversion and electron transfer of polysulfides. Additionally, Ti_4_O_7_ formed strong chemical bonds with polysulfides, effectively mitigating their shuttle effect. Figure 7 illustrates the separator preparation process and demonstrates the exceptional electrochemical performance of the lithium–sulfur battery utilizing this separator. TEM and SEM images reveal the random distribution of Ti_4_O_7_ particles on the fiber’s surface, leading to substantial inhibition of polysulfide shuttling and a notable improvement in the electrochemical performance of lithium–sulfur batteries. At a discharge rate of 0.2 C, the initial specific capacity reached an impressive 1304 mAh g^−1^, with the capacity maintained at 945 mAh g^−1^ after 100 cycles. During high-rate cycling tests at 5 C, the initial specific capacity was 610 mAh g^−1^, and even after 300 cycles, the capacity remained at approximately 400 mAh g^−1^, with a minimal capacity decay of only 0.11% per cycle. Furthermore, in the impedance diagram, the TCNFs interlayer exhibited the lowest AC impedance, measuring only 40 Ω (that of the comparison sample was 138 Ω).

Introducing an electrospun nanofiber interlayer with high electrical conductivity and surface area between the positive electrode and separator serves not only to enhance the conductivity of the integrated electrode, but also to significantly suppress the migration of polysulfides towards the negative electrode side. As shown in Table 1, the application of electrospinning separators and interlayers in lithium–sulfur batteries is demonstrated.

### 4.2. Vacuum Filtration

#### 4.2.1. Separator

**Figure 8:**
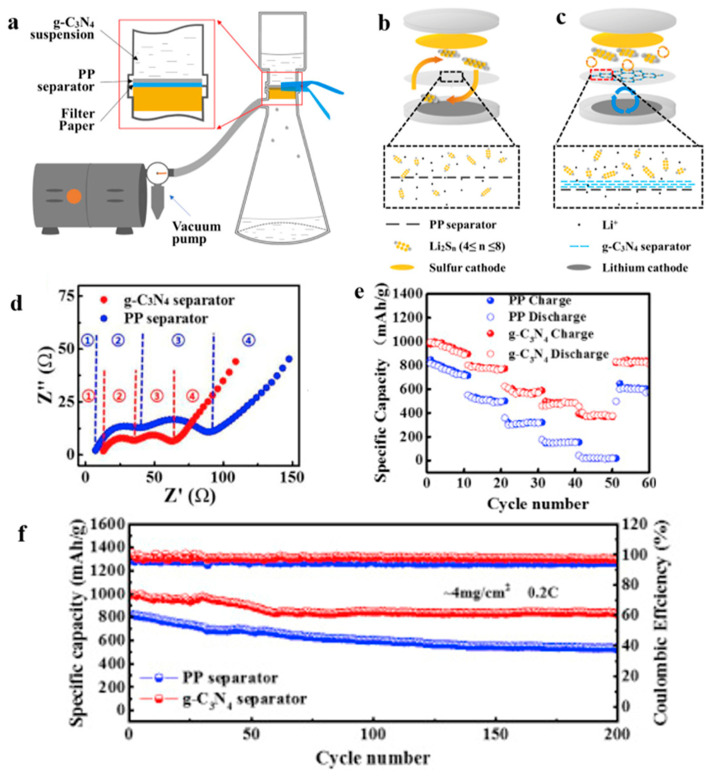
Application of a separator prepared by vacuum filtration method in a lithium–sulfur battery: (**a**) Schematic diagram of separator modification, (**b**) schematic diagram of PP separator barrier to polysulfide, (**c**), schematic diagram of g−C_3_N_4_/PP separator barrier to polysulfide, (**d**) Electrochemical impedance spectroscopy after cycling, (**e**) rate performance, and (**f**) cycle curve [89]. Reproduced with permission from the Electrochimica Acta.

According to Yigeng et al. [89], They modified the PP separator through suction filtration, a depositing a layer of g-C3N4 composite on one side of it. It had abundant adsorption sites and contributed to the solidification of polysulfides. The process of polysulfide solidification entails immobilizing polysulfide species onto the cathode material or the cathode-proximate regions of the separator. This strategic immobilization serves as an effective countermeasure against the undesirable migration of polysulfides towards the anode, thereby mitigating capacity fade and consequentially augmenting the performance characteristics of lithium–sulfur batteries. As shown in Figure 8, the schematic diagram of the suction filter system is shown, and SEM revealed that the PP separator has large pores, that polysulfides are easy to pass through, and that the modified g-C_3_N_4_ separator could not see the large pores of PP. Through electrochemical impedance analysis (EIS), after cycling, it was seen that the modified g-C_3_N_4_ separator had a small impedance. When the discharge current was restored to 0.2 C, the reversible capacity could be restored to 830 mAh g^−1^, indicating that the electrochemical stability of the separator after modification had been significantly enhanced. It can be seen from the cycle performance chart that at 0.2 C, the initial discharge-specific capacity was 990 mAh g^−1^; after 200 cycles, the discharge-specific capacity was 829 mAh g^−1^, and the capacity retention rate was 83.7%.

The vacuum filtration method provides the advantages of high porosity, consistency, controllability, and cost-effectiveness for the preparation of lithium–sulfur battery separators.

#### 4.2.2. Interlayer

**Figure 9:**
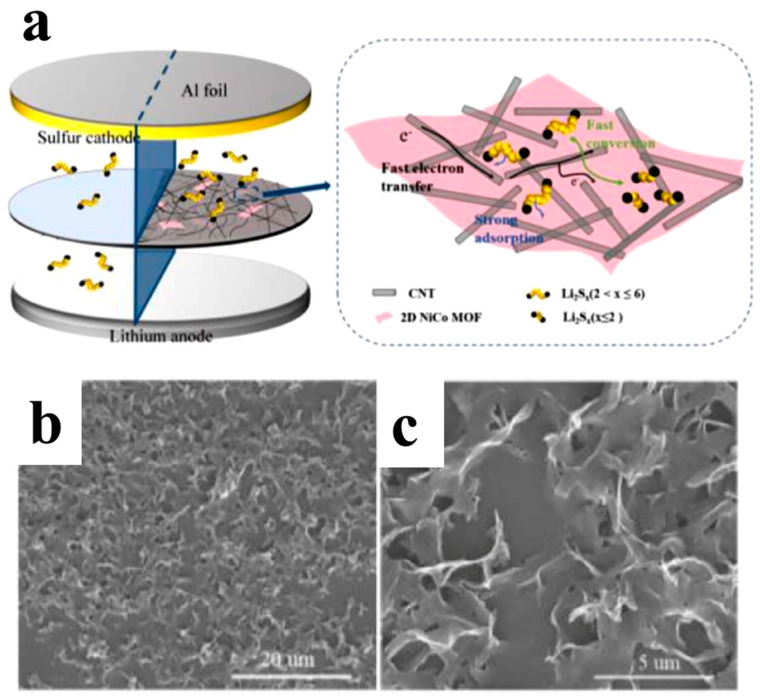
Application of interlayer prepared by vacuum filtration method in a lithium−sulfur battery: (**a**) Internal diagram of the battery, (**b**,**c**) SEM at different magnifications [90]. Reproduced with permission from the Chinese Chemical Letters.

Feng et al. [90] prepared a 2D NiCo MOF/CNT as the middle layer of a lithium–sulfur battery and filtered it onto PP through vacuum filtration. As shown in Figure 9, the thickness of 2D NiCo MOF/CNT was only a few nanometers, and the CNT built a conductive network to enhance electronic conductivity while serving as a physical barrier to prevent polysulfide migration. The 2D NiCo MOF/CNT improved the catalytic performance due to abundant and accessible active sites. The lithium–sulfur battery using 2D NiCo MOF/CNT interlayer had an initial discharge-specific capacity of 1132.7 mAh g^−1^ at 0.5 C, and it maintained 709.1 mAh/g^-1^ after 300 cycles, showing good cycle stability and rate performance.

Whether for the preparation of separators or interlayers, using vacuum filtration provides the advantages of high porosity, consistency, controllability, and cost-effectiveness. Shown in Table 2 is the application of the separator and interlayer prepared by the vacuum filtration method in lithium–sulfur batteries.

### 4.3. Wet Spinning

Currently, commercial PP and PE separators are prepared using a wet process. However, when used directly in lithium–sulfur batteries, the PP separator leads to a significant shuttle effect, resulting in a sharp decrease in the specific discharge capacity of the battery. Therefore, we will not elaborate on the application of PP separators in lithium–sulfur batteries separately in this context. Main applications include functional interlayers in Li-S [18,97,98,99,100] and electrodes [101,102]. Li et al. [98] successfully prepared a carbon paper sandwich via a wet process with excellent electrical conductivity of 11.9 S cm^−1^. A high initial capacity, up to 1091 mAh g^−1^, was achieved when using carbon paper as an interlayer for Li-S. At 5 C, 631 mAh g^−1^ was maintained after 200 cycles (0.21% capacity decay per cycle). Zhang et al. [103]. prepared the PMB-SNWs interlayer through a wet process. The interlayer was applied in lithium–sulfur batteries and exhibited good electrochemical performance. At 2 C, the initial discharge-specific capacity was 877 mAh g^−1^; it maintained 782 mAh g^−1^ after 850 cycles, and the decay rate per cycle was as low as 0.013%.

### 4.4. Coating Method

#### 4.4.1. Separator

**Figure 10:**
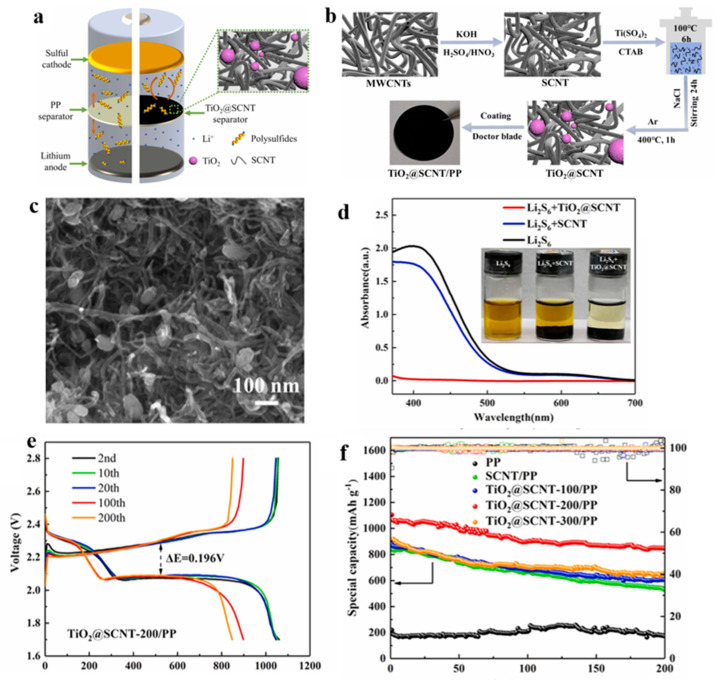
Application of a separator prepared by the coating method in lithium−sulfur batteries: (**a**) schematic diagram of the battery, (**b**) preparation flow chart, (**c**) scanning electron microscopy diagram, (**d**) schematic diagram of the adsorption effect of polysulfide, (**e**) charge and discharge curve, and (**f**) After 200 cycles at 0.5C [57]. Reproduced with permission from the Journal of Alloys and Compounds.

Polar metal oxide titanium dioxide (TiO_2_) exhibits a strong chemical interaction with polysulfides, making it widely applicable in the field of lithium–sulfur batteries. This is attributed to the ability of oxygen atoms on the surface of TiO_2_ to form chemical bonds with sulfur atoms present in polysulfides, facilitating the effective adsorption of polysulfides. Additionally, the high electronegativity of the TiO_2_ surface enables it to counteract the shuttle effect of polysulfides through a charge repulsion mechanism. Gao et al. [57] coated the PP separator with a layer of titanium dioxide, modified multi-walled carbon nanotube composites (TiO_2_@SCNT/PP separator), and applied the separator to Li-S, and the data showed that the performance of the separator and the pre-modification period greatly improved. In Figure 10, the battery’s schematic diagram and the SEM images illustrate the separator preparation process for blocking polysulfides. The SCNTs are intertwined to form a conductive framework that enhances electron transport. TiO2 particles are embedded in SCNT, and have a strong chemical adsorption effect which can inhibit the shuttle effect. By comparing the color changes of polysulfides, it was also shown that the PP separator coated with TiO_2_@SCNT composites had a good adsorption effect on polysulfides. Electrochemical performance is an important measure of the battery, and this separator was assembled in the battery. The data show that the performance was best when the coating thickness was 200, denoted as TiO_2_@SCNT-200/PP, at 0.5 C. The initial discharge specific capacity of the composite separator was 1103.9 mAh g^−1^ (compared to PP separator 218.5 mAh g^−1^); after 200 cycles, the capacity ratio was 848.0 mAh g^−1^ (compared to PP separator is 172.2 mAh g^−1^); and after 900 cycles, it maintained 446.8 mAh g^−1^. The capacity decay per cycle was only 0.066%. The electrochemical performance was greatly improved compared to the PP separator.

#### 4.4.2. Interlayer

**Figure 11:**
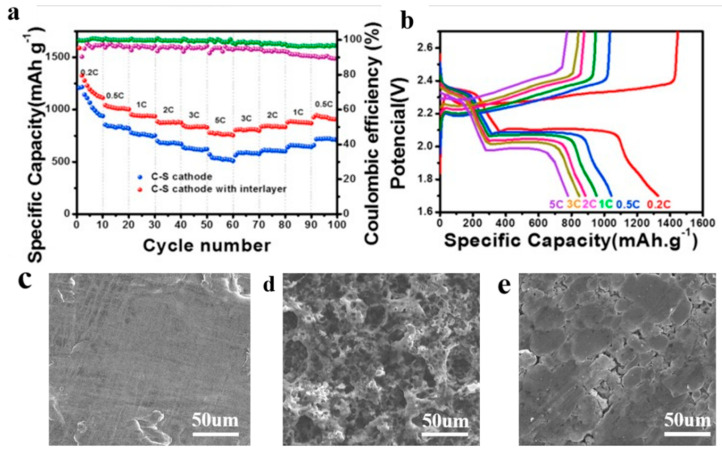
Application of an interlayer prepared by the coating method in lithium−sulfur batteries: (**a**) differences in specific capacity and Coulomb efficiency at various rates, (**b**) its corresponding voltage−capacity profiles at various rates, (**c**) lithium metal surface before cycling, (**d**) lithium metal surface after cycling without interlayer, (**e**) lithium metal surface after cycling with interlayer [104]. Reproduced with permission from the Chemical Engineering Journal.

Wang et al. [104] prepared the NC-Co interlayer using a coating method. The intermediate layer effectively inhibited the shuttling of polysulfides. As shown in Figure 11, at 1 C, the first discharge-specific capacity of the lithium–sulfur battery using the interlayer was 1216.9 mAh g^−1^, and it maintained 660.3 mAh g^−1^ after 250 cycles. The Coulombic efficiency remained above 99% during the cycle. After 100 cycles, the surface SEM of the negative lithium metal showed that the lithium negative electrode with the interlayer had few surface cracks, while the lithium metal without the interlayer had obvious cracks, indicating that the use of the interlayer can significantly inhibit the corrosion of the negative metal lithium.

The coating method can provide structural uniformity, controllability, multiple material options, and cost-effectiveness. These characteristics are very beneficial for the preparation of lithium–sulfur battery separators and interlayers. Shown in Table 3 is the application of separators and interlayers prepared using the coating method in lithium–sulfur batteries.

### 4.5. In Situ Growth Method

#### 4.5.1. Separator

**Figure 12:**
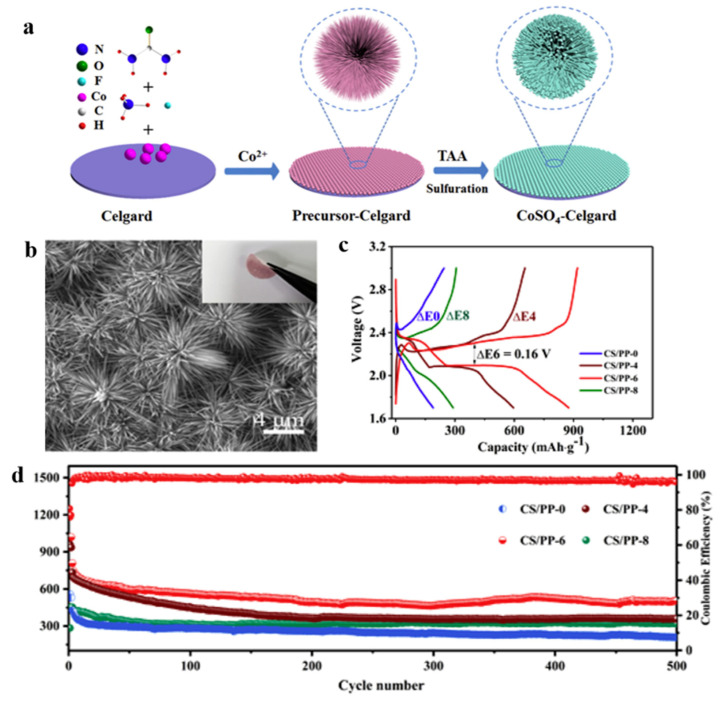
Application of a separator prepared using the original growth method in lithium−sulfur batteries: (**a**) preparation flow chart, (**b**) scanning electron microscopy diagram, (**c**) charge and discharge curve, and (**d**) cycle curve [111]. Reproduced with permission from the Journal of Alloys and Compounds.

Lu et al. [111] modified a PP separator via in situ growth. On the side of the PP separator, a layer of polar hydrated sulfate CoSO_4_·4H_2_O material (CS/PP separator) was grown in situ, and the cobalt sulfate hydrate had strong polarity and catalytic properties, which can effectively adsorb polysulfides. As shown in Figure 12, in the preparation flow chart, it can be seen by scanning electron microscopy that the surface of the separator was attached to a layer similar to the shape of a sea urchin, the single sea urchin was assembled from the nanoneedles of several microns, and the separator exhibited good mechanical stability. The data show that when the material reaction time was 6 h, the separator showed the best performance, which was denoted as CS/PP-6. At 1 C, the initial specific capacity of the modified separator reached as high as 807.7 mAh g^−1^. After 500 cycles, it still maintained 504.6 mAh g^−1^ (compared to 208.7 mAh g^−1^ for the PP separator), and the Coulombic efficiency reached as high as 97%. When the discharge current was restored to 0.1 C, the reversible capacity was 1308.6 mAh g^−1^, indicating that the lithium–sulfur battery using the separator had good reversibility and good electrochemical performance.

#### 4.5.2. Interlayer

**Figure 13:**
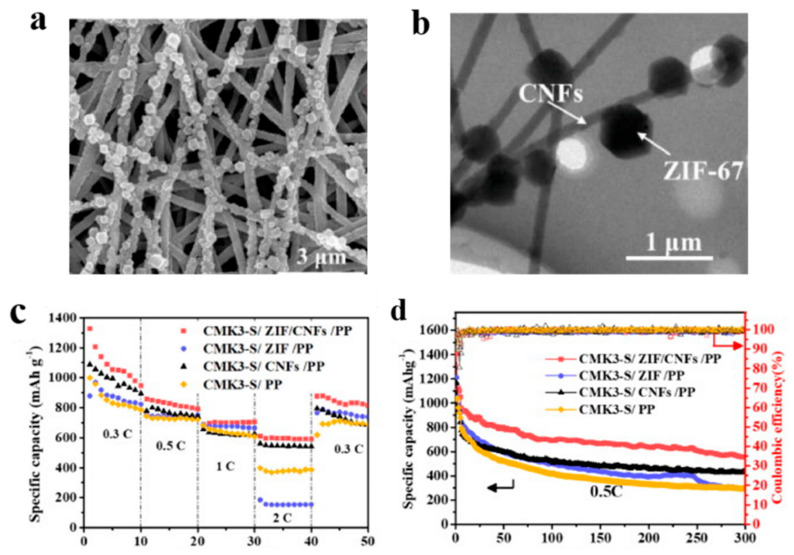
Application of Interlayer Prepared by Original Growth Method in Lithium–sulfur Batteries: (**a**) SEM image of ZIF/CNFs, (**b**) TEM image of ZIF/CNFs, (**c**) Rate performance, (**d**) After 300 cycles at 0.5C [112]. Reproduced with permission from the Journal of Energy Chemistry.

Li et al. [112] prepared the ZIF/CNFs interlayer via the situ growth method. As shown in Figure 13, the interlayer had an obvious effect of inhibiting polysulfide shuttling. According to SEM and TEM images, ZIF-64 particles were distributed on the fiber, and due to the special binding site of ZIF-64, it inhibited the shuttling of polysulfides during circulation. At 1 C, it exhibited a high discharge-specific capacity of 1334 mAh/g, which remained at 569 mAh/g after 300 cycles.

The in-situ growth method has high controllability and nanoscale pore structure in the preparation of lithium–sulfur battery separators and interlayers, which can significantly improve battery performance. As shown in Table 4, the application of separators and interlayers prepared by the situ growth method in lithium–sulfur batteries.

### 4.6. Atomic Layer Deposition

#### 4.6.1. Separator

At present, owing to the distinctive attributes of the Atomic Layer Deposition (ALD) fabrication process, there is a scarcity of literature that directly employs ALD for the modification of polypropylene (PP) separators. Usually, people use methods such as cooperating with other preparation processes (coating, suction filtration, etc.) to modify the separator. Details are shown in Section 4.7.1.

#### 4.6.2. Interlayer

**Figure 14:**
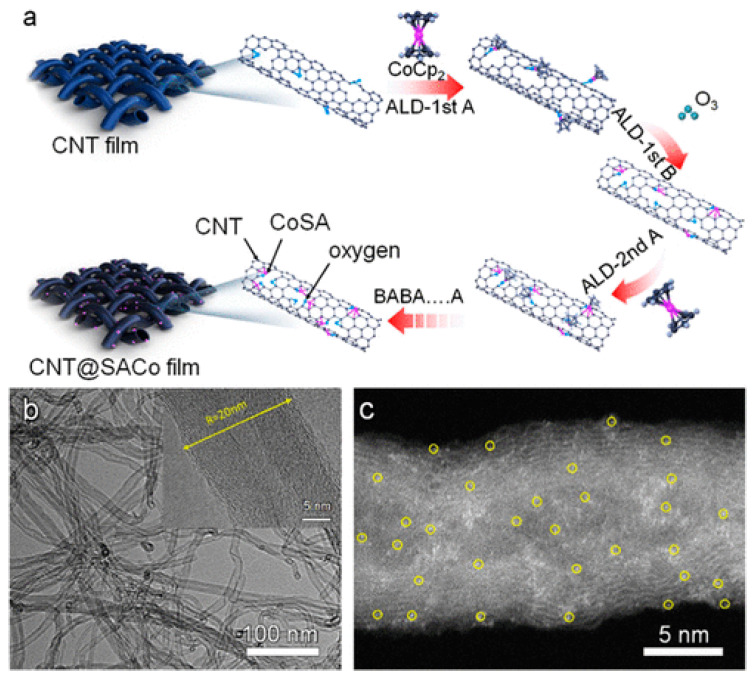
Application of Interlayer Prepared by Atomic Layer Deposition in Lithium–sulfur Batteries: (**a**) Schematic illustration of the preparation process of the CNT@SACo interlayer by the ALD method, (**b**) TEM of CNT@SACo, (**c**) high-angle annular dark-field scanning transmission electron microscopy (HAADF-STEM) image of CNT@SACo [70]. Reproduced with permission from the ACS Applied Energy Materials.

Lin et al. [70]. prepared the CNT@SACo interlayer by atomic layer deposition and applied it to lithium–sulfur batteries. Figure 14 shows the preparation process flow chart. From the experimental results, the CNT@SACo interlayer exhibits catalytic activity catalyzes the conversion of polysulfides, and inhibits the shuttling of polysulfides. The lithium–sulfur battery with the CNT@SACo interlayer exhibited a high discharge specific capacity of 880 mAh/g at 1 C, and maintained a capacity of 595 mAh/g after 500 cycles, with a capacity decay rate of 0.064% per cycle.

The Atomic Layer Deposition method has highly precise film control and uniform material distribution in the preparation of the middle layer of lithium–sulfur batteries, which helps improve battery performance. As shown in Table 5, the application of interlayers prepared by Atomic Layer Deposition (ALD) in lithium–sulfur batteries.

### 4.7. Composite Process

#### 4.7.1. Separator

**Figure 15:**
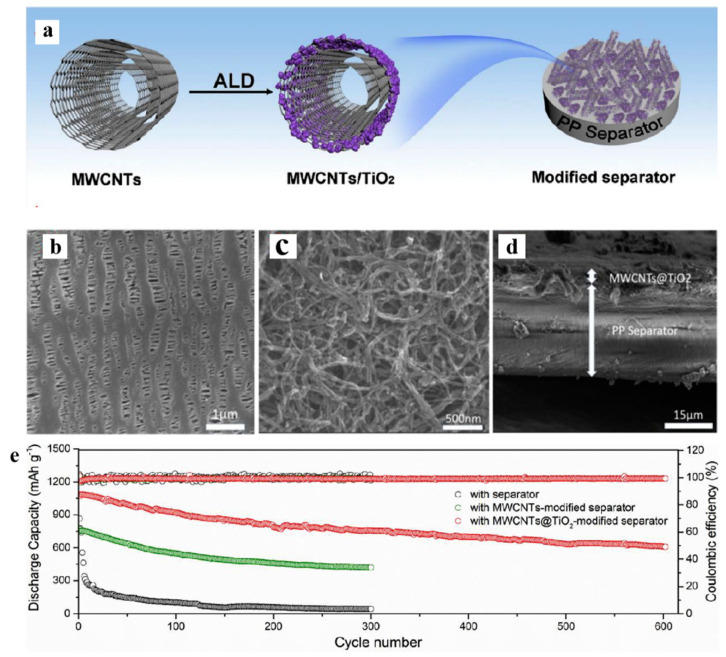
Application of a separator prepared by the composite process in lithium–sulfur batteries: (**a**) schematic diagram of preparation, (**b**) PP separator SEM, (**c**) MWCNTs@TiO_2_/PP separator SEM, (**d**) MWCNTs@TiO_2_/PP separator profile SEM, and (**e**) cycle curve [119]. Reproduced with permission from the Electrochimica Acta.

Ding et al. [119] prepared multi-walled carbon nanotubes @titanium dioxide quantum dots (MWCNTs@TiO_2_) modified PP separators using atomic layer deposition (ALD). Figure 15 shows a flow chart of the preparation process. It can be seen from scanning electron microscopy that the initial PP separator had a large pore size, and the MWCNTs@TiO_2_ was intertwined after modification. The porosity increased and the pore size was improved, effectively preventing soluble polysulfides from shuttling into the anode through the separator. The electrochemical data of the cell showed that the Coulombic efficiency and cycle stability of Li-S cells were improved. At 0.5 C, the capacity decay of Li-S using this separator was reduced to 0.072% per cycle.

#### 4.7.2. Interlayer

**Figure 16:**
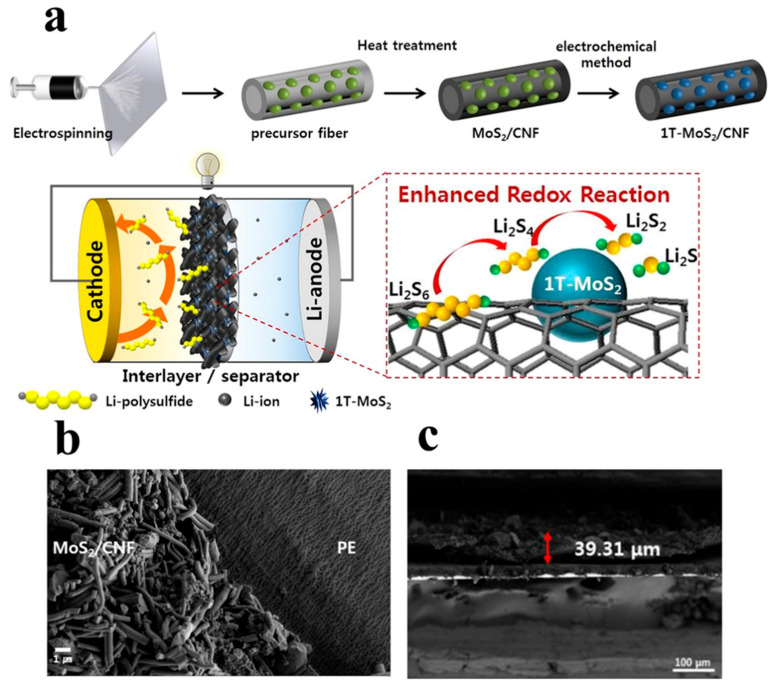
Application of an interlayer prepared using the composite process in lithium–sulfur batteries: (**a**) schematic diagram of preparation, (**b**) SEM, (**c**) TEM [120]. Reproduced with permission from the Journal of Alloys and Compounds.

Sang-Hyun Moon et al. [120] used a combination of electrospinning and vacuum filtration to prepare a 1T-MoS_2_/CNF intermediate layer and applied it to lithium–sulfur batteries. As shown in Figure 16, carbon nanofibers have strong conductivity and can effectively reduce interface resistance. MoS_2_ can strongly adsorb the Li_2_S_x_ due to interactions of S-S and metal-S bonds, preventing the dissolution of Li-polysulfide in a liquid electrolyte. When the interlayer is applied to lithium–sulfur batteries, it exhibits good electrochemical performance. After 500 cycles at 1 C, the capacity retention rate was 73% under 1C. The initial specific capacity reached as high as 480 mAh g^−1^.

Shown in Table 6 is the application of interlayers prepared by the composite process in lithium–sulfur batteries.

## 5. Conclusions and Outlook

In recent years, with the rising demand for new energy, it has become important to develop an energy storage system with high energy density, low cost, and a long cycle life. Traditional lithium batteries have a high cost and low energy density, which makes it difficult for them to meet the huge market demand. Li-S batteries are regarded as one of the most promising energy storage systems due to their high theoretical specific capacity and low cost. Li-S batteries also have some urgent problems to solve, such as poor conductivity of S, the expansion of the positive electrode volume during the electrochemical reaction, and the most important problem, the shuttle effect caused by polysulfides. As an important component of lithium–sulfur batteries, separators are very important in suppressing the shuttle effect of polysulfides. 

From the perspective of separator preparation, the research mainly focuses on the preparation of new separator materials and the modification of traditional separators. Due to their high porosity and liquid absorption rates, electrospinning technology can effectively enhance the actual discharge-specific capacity of lithium–sulfur batteries. However, the electrospun fiber separator tends to have a relatively large thickness and low mechanical strength. In this field, future advancements can be made by starting at the raw material level to develop high-performance electrospun nanofibers that exhibit excellent strength and temperature resistance. For instance, the PI fiber demonstrates exceptional temperature resistance, along with favorable mechanical properties and other new electrospinning separation materials, including PAN, polyvinyl chloride (PVC), PVDF, and polyethylene oxide (PEO) separators.

Lithium–sulfur batteries have undergone significant progress; however, most research is still based on cylindrical cell designs. The real operational environment for pouch-type cells is more demanding due to their lower sulfur active material content, making it challenging to achieve high energy density. Additionally, the fabrication processes for modified separators and interlayers are currently not scalable and come with higher costs. Through the optimization of separator structures and the exploration of new materials, there is potential to further enhance the electrochemical performance of lithium–sulfur batteries while reducing the costs. Some perspectives regarding the future of lithium–sulfur battery separators in terms of structural design and material selection are listed as follows: (1) ion-selective separators: This type of separator can block the shuttling of polysulfides while allowing for the passage of lithium ions. Implementing ion-selective separators holds the promise of improving Coulombic efficiency and battery cycle life. (2) Multilayer structure separators: Multilayer separators composed of layers with different properties can achieve more precise control over the shuttling of polysulfides while providing higher ion transport rates. (3) Porous separators: Porous separators with high porosity can accommodate more electrolytes, contributing to improved electrical conductivity and polysulfide adsorption capability in lithium–sulfur batteries. Researchers are exploring various pore sizes and pore structures to optimize battery performance. (4) Metal–organic framework (MOF) materials: MOFs, known for their large surface areas and porous structures, have found applications in lithium–sulfur battery separators due to their exceptional ability to adsorb polysulfides. (5) Biobased materials: Biodegradable materials, with their natural resources, renewability, and environmental friendliness, hold potential for use in eco-friendly batteries. (6) Two-dimensional materials: Two-dimensional materials such as graphene have been investigated for use in battery separators due to their excellent conductivity and mechanical strength, which could enhance battery performance. These innovative structural designs and material selections are expected to drive the commercialization of lithium–sulfur batteries in the future, playing a crucial role in energy storage systems.

## Figures and Tables

**Table 1:** Applications of electrospun separators and interlayers in lithium–sulfur batteries.

	Main Materials	Initial Capacity (mAh g^−1^)	Capacity Remaining (mAh g^−1^)	Decay Rate	SEM Figure	Reference
Separator	PVDF/PSSLi	955	466 (0.5 C, 200 cycles)	0.26%	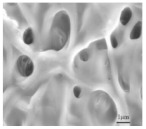	[82]
	PVDF/MOF	1324.2	551 (2 C, 700 cycles)	0.05%	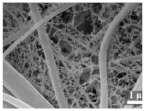	[83]
	PI/MC	1602.3	905.5 (0.2 C, 100 cycles)	---	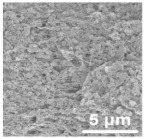	[84]
	PMIA	1222.25	745.7 (0.5 C, 800 cycles)	---	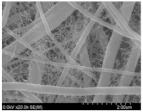	[85]
Interlayer	TMN@CNF	947	390 (2 C, 1000 cycles)	0.059%	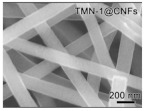	[86]
	TCNF	1279	798 (2.5 A g^−1^, 1000 cycles)	0.057%	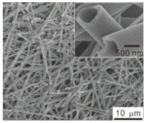	[87]
	CoSe@NC	1317	804.7 (0.1 C, 100 cycles)	---	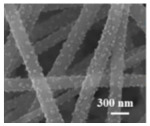	[88]

**Table 2:** Applications of vacuum filtration separators and interlayers in lithium–sulfur batteries.

	Main Materials	Initial Capacity (mAh g^−1^)	Capacity Remaining (mAh g^−1^)	Decay Rate	SEM Figure	Reference
Separator	BC	1175	735 (0.3 C, 300 cycles)	0.07%	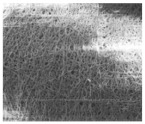	[91]
	HCNF/rGO	1318.4	533.6 (1 C, 400 cycles)	0.13%	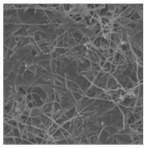	[92]
	CF/PP	1111	683 (0.5 C, 500 cycles)	0.071%	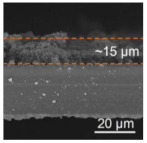	[93]
	NiFe_2_O_4_–NiO/PP	1350	755 (2 C, 1000 cycles)	0.065%	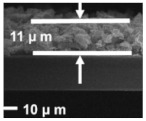	[94]
Interlayer	MOF	850	604 (1 C, 900 cycles)	0.032%	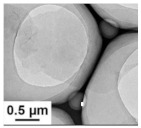	[95]
	Co-MOF-74@MWCNT	1434	771 (0.1 C, 200 cycles)	---	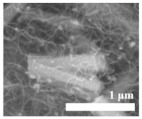	[96]

**Table 3:** Applications of separators and interlayers prepared using the coating method in lithium–sulfur batteries.

	Main Materials	Initial Capacity (mAh g^−1^)	Capacity Remaining (mAh g^−1^)	Decay Rate	SEM Figure	Reference
Separator	Al_2_O_3_/PP	967	593.4 (0.5 C, 50 cycles)	0.13%	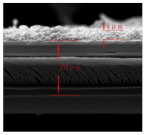	[105]
	Zr-MOF@CNT/PP	1157	545 (1 C, 500 cycles)	0.067%	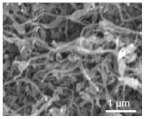	[106]
	ZIF-67/PP	1341	761 (0.2 C, 300 cycles)	0.14%	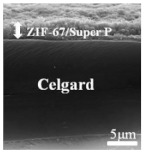	[107]
	RP/PP	1287	729.6 (1 C, 500 cycles)	0.109%	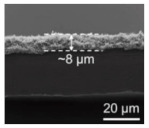	[108]
Interlayer	CFF	1346.9	1076.6 (0.1 C, 100 cycles)	---	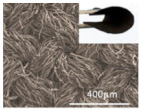	[109]
	OMNC	994.4	587.6 (0.5 C, 100 cycles)	---	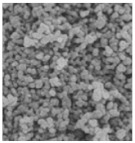	[110]

**Table 4:** Applications of the situ growth method separators and interlayers in lithium–sulfur batteries.

	Main Materials	Initial Capacity (mAh g^−1^)	Capacity Remaining (mAh g^−1^)	Decay Rate	SEM Figure	Reference
Separator	TA-Co/PP	1182	549.9 (2 C, 500 cycles)	0.065%	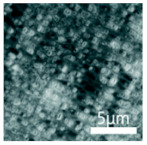	[69]
	Z-PMIA	1391.2	961.1 (0.2 C, 350 cycles)	0.033%	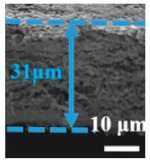	[55]
	PMIA/ZIF-8	1156	855 (0.2 C, 300 cycles)	0.086%	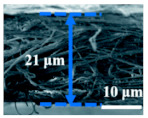	[113]
Interlayer	MIL-101/CNT	816	628 (1 C, 500 cycles)	0.046%	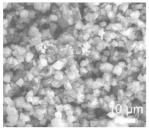	[114]
	MOF-808	908.1	755.5 (1 C, 500 cycle)	0.03%	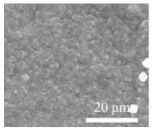	[115]

**Table 5:** Applications of Atomic layer deposition (ALD) separators and interlayers in lithium–sulfur batteries.

	Main Materials	Initial Capacity (mAh g^−1^)	Capacity Remaining (mAh g^−1^)	Decay Rate	SEM Figure	Reference
Interlayer	ALD-ZnO	998	846 (0.2 C,100 cycles)	---	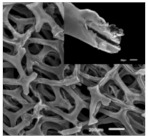	[116]
	Al_2_O_3_	1136	766 (40 cycles)	---	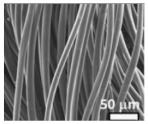	[117]
	HfO_2_-CNT	1275	995 (0.2 C, 100 cycles)	---	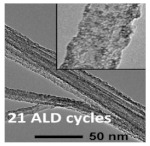	[118]

**Table 6:** Applications of composite process separators and interlayers in lithium–sulfur batteries.

	Main Materials	Initial Capacity (mAh g^−1^)	Capacity Remaining (mAh g^−1^)	Decay Rate	SEM Figure	Reference
Separator	Ni-Co MOF@PAN	944	794 (2 C, 2000 cycles)	0.034%	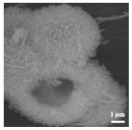	[121]
	PAN/PDAAQ	881	766 (1 C, 800 cycles)	0.11%	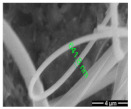	[122]
	UIO66@BP/PAN	---	761 (0.5 C, 1000 cycles)	0.016%	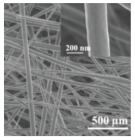	[123]
Interlayer	N, Co-TiO x/NCNT@CNFs	1132	988 (0.2 C, 100 cycles)	---	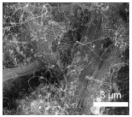	[124]
	CNF@VS 2/CNT	834	605 (1 C, 1145 cycles)	---	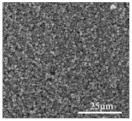	[125]

## Data Availability

Not applicable.
